# Supplementary material for: Association between Early Phase Serum Albumin Levels and Outcomes of Post-Cardiac Arrest Patients: A Systematic Review and Meta-Analysis
Source: J Pers Med. 2022 Oct 29;12(11):1787. doi: 10.3390/jpm12111787 (PMC9697792; doi:10.3390/jpm12111787)
Supplement: Supplementary file 1 [file jpm-12-01787-s001.zip › jpm-1871803-supplementary.pdf]

Table S1. Comprehensive list presenting the search strategy.

EMBASE (1974 to 2022 July 01)

1	(return of spontaneous circulation or ROSC).ti,ab. or exp heart arrest/ or cardiac arrest*.ti,ab. or cardiovascular arrest*.ti,ab. or heart arrest*.ti,ab. or cardiopulmonary arrest*.ti,ab. or asystol*.ti,ab. or pulseless electrical activity.ti,ab. or exp ventricular fibrillation/ or exp advanced cardiac life support/ or (advanced cardiac life support or ACLS).ti,ab. or exp cardiopulmonary resuscitation/ or CPR.mp. or cardiopulmonary resuscitation.ti,ab.	240736
2	albumin.ti,ab.	30624
3	1 and 2	252

MEDLINE (1946 to July 01, 2022)

1	(return of spontaneous circulation or ROSC).ti,ab. or exp heart arrest/ or cardiac arrest*.ti,ab. or cardiovascular arrest*.ti,ab. or heart arrest*.ti,ab. or cardiopulmonary arrest*.ti,ab. or asystol*.ti,ab. or pulseless electrical activity.ti,ab. or exp ventricular fibrillation/ or exp advanced cardiac life support/ or (advanced cardiac life support or ACLS).ti,ab. or exp cardiopulmonary resuscitation/ or CPR.mp. or cardiopulmonary resuscitation.ti,ab.	103830
2	album.ti,ab.	25408
3	1 and 2	36

Cochrane

1	MeSH descriptor: [Return of Spontaneous Circulation] explode all trees	5
2	MeSH descriptor: [Heart Arrest] explode all trees	2159
3	MeSH descriptor: [Advanced Cardiac Life Support] explode all trees	62
4	MeSH descriptor: [Resuscitation] explode all trees	5625
5	#1 or #2 or #3 or #4	7003
6	MeSH descriptor: [Albumins] explode all trees	8360
	#5 and #6	80

Table S2. Characteristics of excluded from meta-analysis.

	Author	Year	Title	Reason for exclusion
1	Matsuyama	2018	Prognostic Impact of Serum Albumin Concentration for Neurologically Favorable Outcome in Patients Treated with Targeted Temperature Management After Out-of-Hospital Cardiac Arrest: A Multicenter Prospective Study.	Irrelevant outcome measure
2	Matsuyama	2018	Effect of Serum Albumin Concentration on Neurological Outcome After Out-of-Hospital Cardiac Arrest.	Irrelevant outcome measure
3	Park	2012	Prognostic significance of serum albumin on long-term mortality in survivors from out-of-hospital cardiac arrest.	Irrelevant outcome measure
4	Kong	2020	The Prognostic Usefulness of the Lactate/Albumin Ratio for Predicting Clinical Outcomes in Out-of-Hospital Cardiac Arrest: A Prospective, Multicenter Observational Study (koCARC) Study.	Duplicate Data
5	Yoon	2020	Effect of serum albumin level on hospital outcomes in out-of-hospital cardiac arrest.	Irrelevant outcome measure

Table S3. Baseline characteristics of individuals in the included studies.

Author	Outcome	Sample size, n	OHCA, %	Age	Male, %	Witness, %	Bystander CPR, %	Shockable rhythm, %	TTM, %
Bingol Tanriverdi	Survivors	43	100	60.3 ± 11.9	67.4	-	-	-	-
	Non-survivors	59	100	61.5 ± 13.0	76.3	-	-	-	-
Kim	GNO	84	100	48.3 ± 15.2	76.2	85.7	61.9	81.0	100
	PNO	171	100	57.9 ± 16.5	67.8	64.7	52.9	17.5	100
Kokulu	Survivors	42	100	62 ± 11	66.7	76.2	54.8	61.9	64.2
	Non-survivors	193	100	68 ± 12	57.5	57.5	43.0	26.4	24.3
Lee	Survivors	204	100	60.7 ± 17.2	71.1	79.9	37.7	31.9	47.1
	Non-survivors	485	100	65.1 ± 17.3	69.9	70.1	44.9	15.9	32.4
	GNO	81	100	56.7 ± 16.9	69.1	88.9	37.0	49.4	35.8
	PNO	608	100	64.7 ± 17.2	70.4	70.9	43.6	16.8	36.8
Li	Overall	2414	-	64 (53-74)	57.1	-	-	-	-
	Survivors	1065	-	63 (52-73)	59.0	-	-	-	-
	Non-survivors	1349	-	66 (55-75)	55.7	-	-	-	-
Son	Overall	59	100	57.8 (52.9-62.7)	72.9	76.3	-	23.7	69.5
	GNO	13	100	48.9 (39.6-58.3)	46.2	92.3	-	61.5	76.9
	PNO	46	100	60.3 (54.6-66.0)	80.4	71.7	-	13.0	67.4
You	Overall	83	-	52.0 (42.0-68.0)	71.1	66.3	44.6	34.9	100
	GNO	28	-	44.0 (30.5-51.5)	82.1	75.0	57.1	46.4	100
	PNO	55	-	60.0 (47.0-73.0)	65.5	61.8	38.2	29.1	100

Table S4. Detailed quality assessment of included studies using the Quality of Prognosis Studies in Systematic Reviews (QUIPS).

**Biases**	**Study number**						
	**1**	**2**	**3**	**4**	**5**	**6**	**7**
	Rating of reporting (yes, partial, no, unsure) and "Risk of bias" (high, moderate, low)						
**Study Participation**							
Source of target population	Y	Y	Y	Y	Y	Y	Y
Method used to identify population	Y	Y	Y	Y	Y	Y	Y
Recruitment period	P	Y	Y	Y	Y	Y	Y
Place of recruitment	Y	Y	Y	Y	Y	Y	Y
Inclusion and exclusion criteria	Y	Y	Y	Y	Y	Y	Y
Adequate study participation	Y	Y	Y	Y	Y	Y	Y
Baseline characteristics	Y	Y	Y	Y	Y	Y	Y
**Summary Study participation**	L	L	L	L	L	L	L
**Study Attrition**							
Proportion of baseline sample available for analysis	N	Y	Y	Y	Y	Y	Y
Attempts to collect information on participants who dropped out	P	Y	U	Y	Y	U	Y
Reasons and potential impact of subjects lost to follow-up	N	U	U	Y	Y	Y	Y
Outcome and prognostic factor information on those lost to follow-up	N	N	N	Y	Y	N	Y
**Study Attrition Summary**	H	H	H	L	L	H	L
**Prognostic Factor Measurement**							
Definition of the PF	Y	Y	Y	Y	Y	Y	Y
Valid and Reliable Measurement of PF	Y	Y	Y	Y	Y	Y	Y
Method and Setting of PF Measurement	Y	Y	Y	Y	Y	Y	Y
Proportion of data on PF available for analysis	Y	Y	Y	Y	Y	Y	Y
Method used for missing data	P	P	P	P	Y	P	Y
**PF Measurement Summary**	L	L	L	L	L	L	L

Modified from: Hayden JA, Côté P, Bombardier C. Evaluation of the Quality of Prognosis Studies in Systematic Reviews. Annals of Internal Medicine. 2006;144:427-437, with the assistance of the QUIPS-LBP Working Group.

Abbreviations: Y, yes; P, partial; N, no; U, unsure; H, high; M, moderate; L, low

Table S4. (Cont.)

Biases	Study number						
	1	2	3	4	5	6	7
**Outcome Measurement**							
Definition of the Outcome	Y	Y	Y	Y	Y	Y	Y
Valid and Reliable Measurement of Outcome	Y	Y	Y	Y	Y	Y	Y
Method and Setting of Outcome Measurement	P	Y	Y	Y	Y	P	Y
**Outcome Measurement Summary**	L	L	L	L	L	L	L
**Study Confounding**							
Important Confounders Measured	U	Y	Y	P	Y	Y	Y
Definition of the confounding factor	N	Y	P	U	N	Y	U
Valid and Reliable Measurement of Confounders	N	Y	Y	P	N	Y	Y
Method and Setting of Confounding Measurement	Y	Y	Y	Y	N	Y	Y
Method used for missing data	N	N	P	U	P	P	Y
Appropriate Accounting for Confounding	P	Y	Y	P	N	Y	Y
**Study Confounding Summary**	H	L	L	H	H	L	L
**Statistical Analysis and Reporting**							
Presentation of analytical strategy	Y	Y	Y	Y	Y	Y	Y
Model development strategy	Y	Y	Y	Y	Y	Y	Y
Reporting of results	Y	Y	Y	Y	Y	Y	Y
**Statistical Analysis and Presentation Summary**	L	L	L	L	L	L	L

Modified from: Hayden JA, Côté P, Bombardier C. Evaluation of the Quality of Prognosis Studies in Systematic Reviews. Annals of Internal Medicine. 2006;144:427-437, with the assistance of the QUIPS-LBP Working Group.

Abbreviations: Y, yes; P, partial; N, no; U, unsure; H, high; M, moderate; L, low

References

1. Bingol Tanriverdi, T.; Patmano, G.; Bozkurt, F.T.; Kaya, B.C.; Tercan, M. Prognostic value of C-reactive protein to albumin ratio in patients resuscitated from out-of-hospital cardiac arrest. *Int. J. Clin. Pr.* **2021**, *75*, e14227, https://doi.org/10.1111/ijcp.14227

2. Kim, S.H.; Youn, C.S.; Kim, H.J.; Choi, S.P. Prognostic value of serum albumin at admission for neurologic outcome with targeted temperature management after cardiac arrest. *Emerg. Med. Int*. **2019**, *2019*, 6132542. https://doi.org/10.1155/2019/6132542

3. Kokulu, K.; Sert, E.T. The role of the lactate/albumin ratio in predicting survival outcomes in patients resuscitated after out-of-hospital cardiac arrest: A preliminary report. *Am. J. Emerg. Med.* **2021**, *50*, 670–674, https://doi.org/10.1016/j.ajem.2021.09.059

4. Lee, J.; Lee, H.; Oh, J.; Lim, T.H.; Kang, H.; Ko, B.S.; Cho, Y.; The Korean Cardiac Arrest Research Consortium KoCARC Investigators. Association between Initial Serum cholesterol Levels and Outcomes of Patients Hospitalized after Out-of-Hospital Cardiac Arrest: A Retrospective Multicenter Registry Study. *J. Pers. Med.* **2022**, *12*, 233. https://doi.org/10.3390/jpm12020233

5. Li, Y.; She, Y.; Mo, W.; Jin, B.; Xiang, W.; Luo, L. Albumin level at admission to the Intensive Care Unit is associated with prognosis in cardiac arrest patients. *Cureus* **2021**, *13*, https://doi.org/10.7759/cureus.14501

6. Son, Y.S.; Kim, K.S.; Suh, G.J.; Kwon, W.Y.; Park, M.J.; Ko, J.I.; Kim, T. Admission levels of high-density lipoprotein and apolipoprotein A-1 are associated with the neurologic outcome in patients with out-of-hospital cardiac arrest. *Clin. Exp. Emerg. Med.* **2017**, *4*, 232–237, https://doi.org/10.15441/ceem.16.164

7. You, Y.; Park, J.; Min, J.; Yoo, I.; Jeong, W.; Cho, Y.; Ryu, S.; Lee, J.; Kim, S.; Cho, S.; et al. Relationship between time related serum albumin concentration, optic nerve sheath diameter, cerebrospinal fluid pressure, and neurological prognosis in cardiac arrest survivors. *Resuscitation* **2018**, *131*, 42–47. https://doi.org/10.1016/j.resuscitation.2018.08.003

Table S5. Sensitivity analysis with prognostic value of serum albumin level for predicting poor neurologic outcome in post-cardiac arrest patients.

Study	ONSD, SMD (95% CI)	p-value	I^2, %	p-value for heterogeneity
All	1.01 [0.49, 1.52]	< 0.001	87	< 0.001
Omitting Kim 2019	1.01 [0.24, 1.79]	0.01	89	< 0.001
Omitting Lee 2022	1.20 [0.46, 1.95]	0.002	87	0.0004
Omitting Son 2017	0.74 [0.35, 1.13]	< 0.001	79	0.008
Omitting You 2018	1.20 [0.54, 1.85]	< 0.001	91	< 0.001

Table S6. Evidence profile of main results using GRADE.

Result	Quality assessment							
	Participants (studies)	Design	Risk of bias	Inconsistency	Indirectness	Imprecision	Publication bias	Overall quality of evidence
Albumin for predicting survival outcome	3440 (4)	Observational studies	No serious	No serious	No serious	No serious	Undetected	Low
Albumin for predicting neurologic outcome	2027 (4)	Observational studies	No serious	No serious	No serious	No serious	Undetected	Low
Result	Summary of findings							Importance
	No of patients		Effect					
	Survival	Non-survival	Relative	Absolute				
Albumin for predicting survival outcome	1354	2086		SMD 0.55 (0.48-0.62)				Critical
	No of patients		Effect					
	Good neurologic outcome	Poor neurologic outcome	Relative	Absolute				
Albumin for predicting neurologic outcome	206	880		SMD 1.01 (0.19-1.52)				Critical

Abbreviations: GRADE, The Grading of Recommendations Assessment, Development and Evaluation; SMD, standardized mean difference

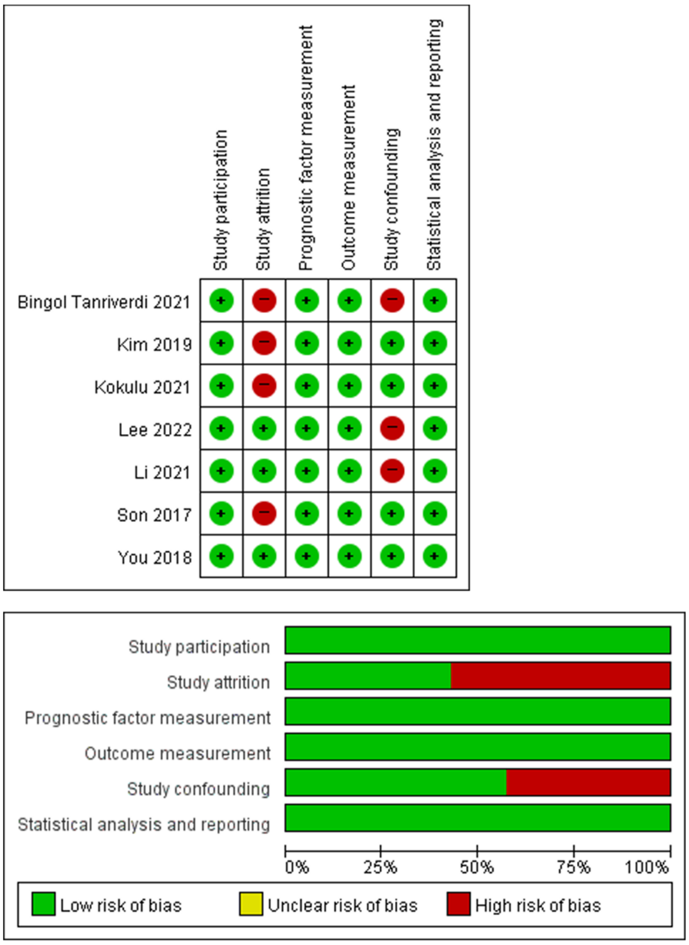

Figure S1. Risk of bias assessment. Author's judgments about each risk of bias item for each included study

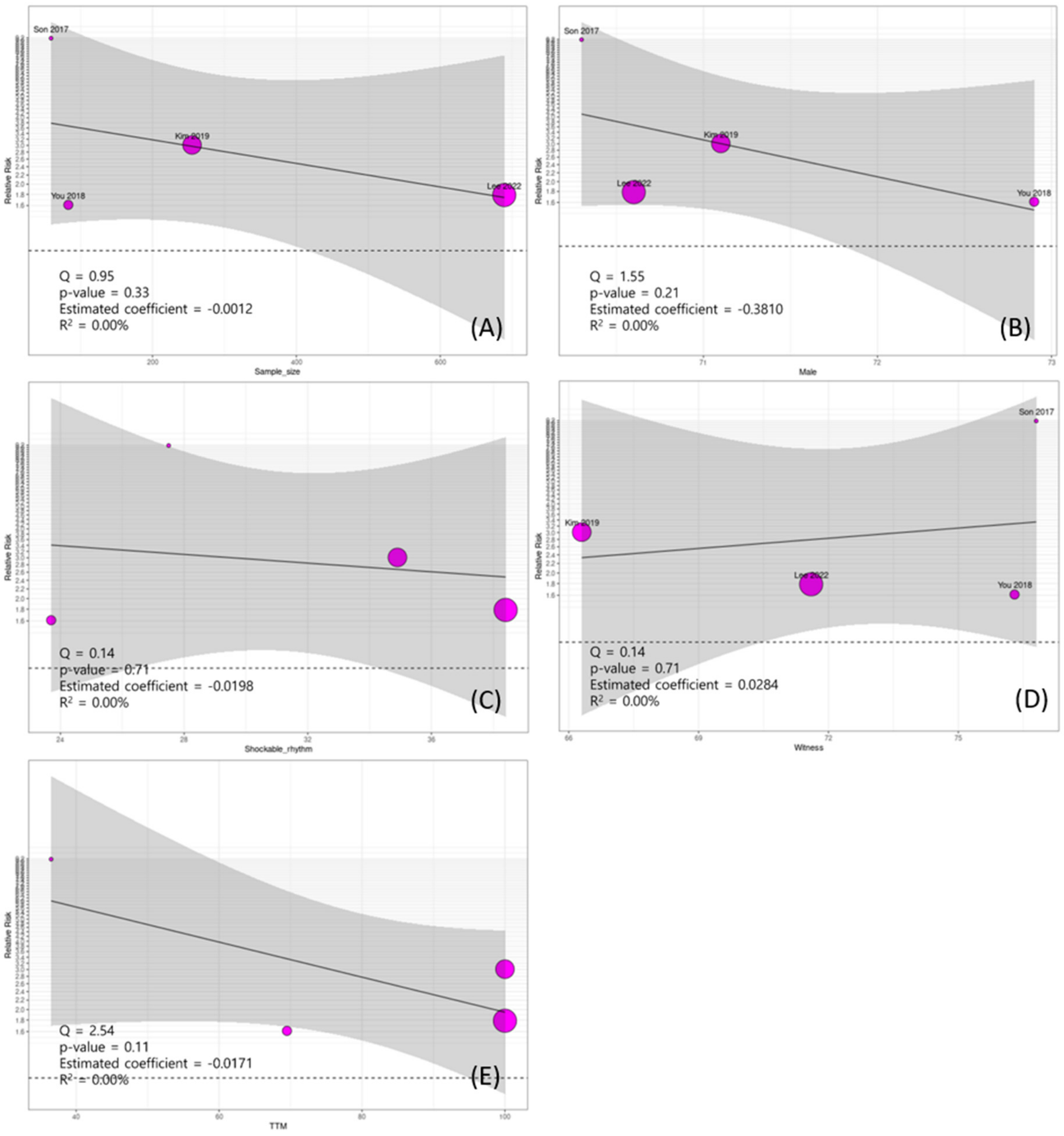

Figure S2. Mixed-effects model meta-regression analysis: Association between the initial albumin level and neurologic outcome in post-cardiac arrest survivors determined using (A) sample size, (B) proportion of male gender, (C) initial shockable rhythm (D) witness arrest, and (E) proportion of targeted temperature management regression.

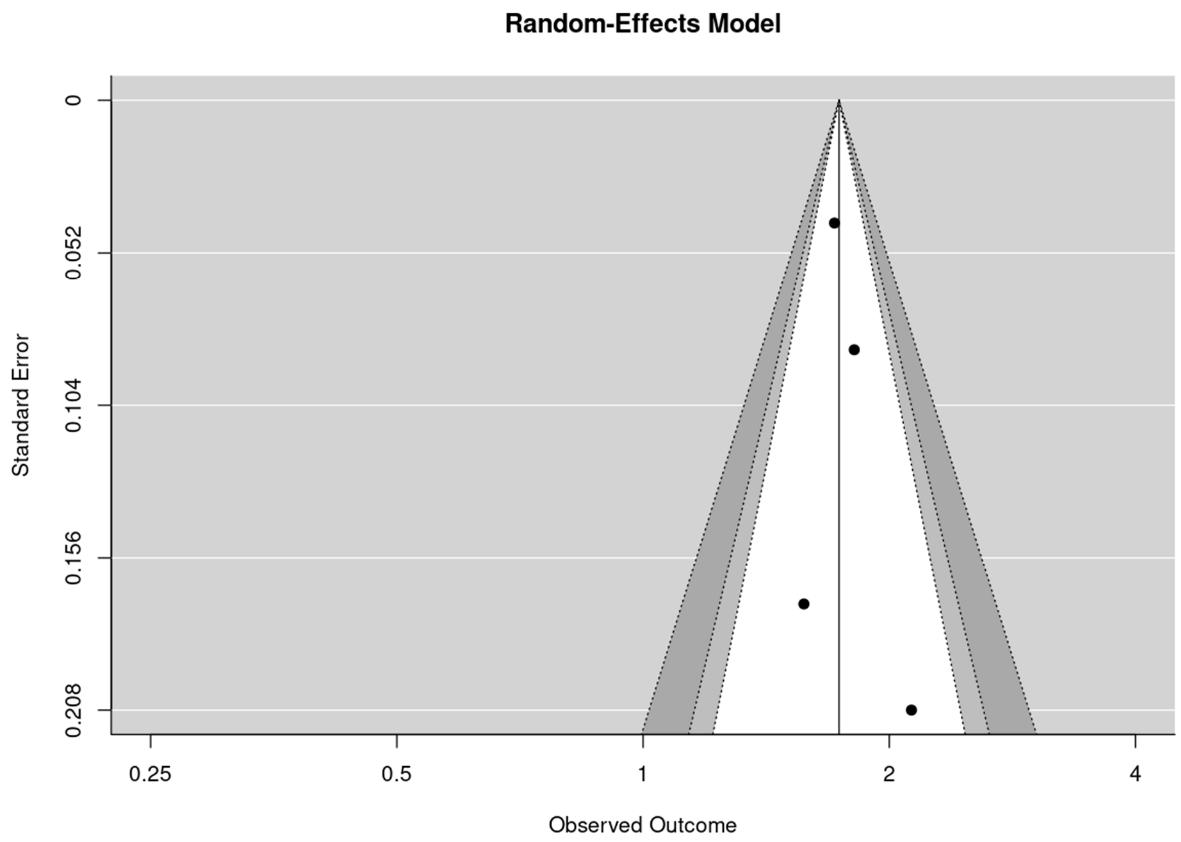
Random-Effects Model
Standard Error
0
0.052
0.104
0.156
0.208
0.25
0.5
1
2
4
Observed Outcome
(A)

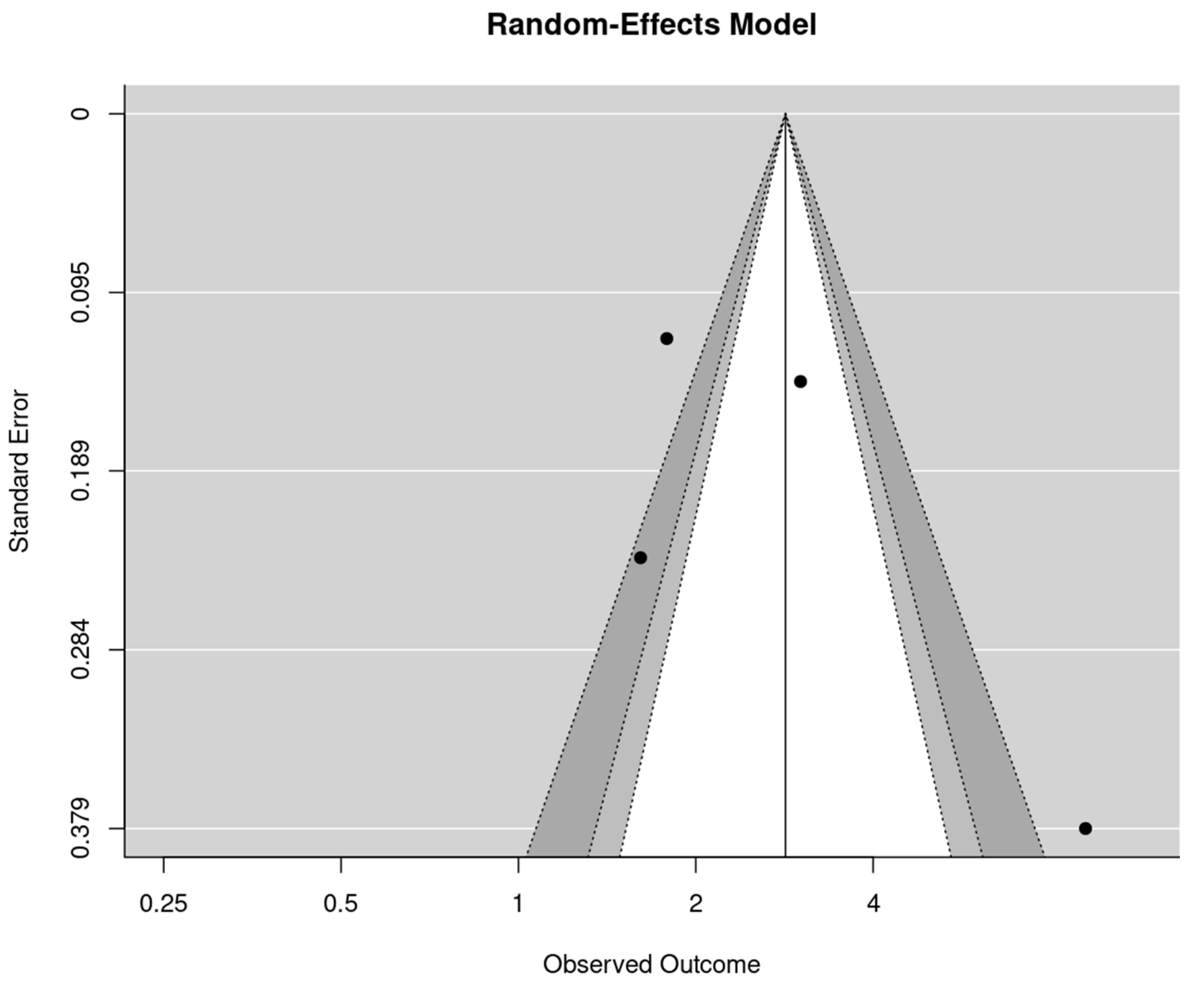

(**B**)

Figure S3 (**A**) Funnel plot to assess the publication bias for the association between serum albumin level and in-hospital mortality in post-cardiac arrest patients**.** (**B**) Funnel plot to assess the publication bias for the association between serum albumin level and neurologic outcome in post-cardiac arrest patients.
